# Management of Donor-Specific Antibodies in Haploidentical Transplant: Multicenter Experience From the Madrid Group of Hematopoietic Transplant

**DOI:** 10.3389/fimmu.2021.674658

**Published:** 2021-05-19

**Authors:** Rebeca Bailén, José Luis Vicario, Laura Solán, Irene Sánchez-Vadillo, Pilar Herrera, María Calbacho, Raquel Alenda, José Luis López-Lorenzo, Karem Humala, Anabelle Chinea, José Sánchez-Pina, Antonio Balas, Miguel Ángel Moreno, Javier Arzuaga, Virginia Pradillo, Nieves Dorado, Gillen Oarbeascoa, Javier Anguita, José Luis Díez-Martín, Mi Kwon

**Affiliations:** ^1^Department of Hematology and Hemotherapy, Hospital General Universitario Gregorio Marañón, Madrid, Spain; ^2^Translational Oncology Section, Gregorio Marañón Health Research Institute, Madrid, Spain; ^3^Department of Histocompatibility, Centro de Transfusión de la Comunidad de Madrid, Madrid, Spain; ^4^Department of Hematology and Hemotherapy, Hospital Universitario Fundación Jiménez Díaz, Madrid, Spain; ^5^Department of Hematology and Hemotherapy, Hospital Universitario La Paz, Madrid, Spain; ^6^Department of Hematology and Hemotherapy, Hospital Universitario Ramón y Cajal, Madrid, Spain; ^7^Department of Hematology and Hemotherapy, Hospital Universitario 12 de Octubre, Madrid, Spain; ^8^Department of Medicine, Universidad Complutense de Madrid, Madrid, Spain

**Keywords:** donor-specific anti HLA antibodies, haplo identical hematopoietic stem cell transplantation, Luminex ^®^, desensitization therapy, kinetics

## Abstract

**Background:**

Donor specific antibodies (DSAs) can be responsible for graft failure (GF) in the setting of mismatched hematopoietic stem cell transplantation (HSCT). The aim of our study is to report the experience of the Madrid Group of Hematopoietic Transplant (GMTH) in patients with DSAs undergoing haplo-HSCT.

**Methods:**

Patients undergoing haplo-HSCT in centers from the GMTH from 2012 to 2020 were included in the study. DSAs were analyzed with a solid-phase single-antigen immunoassay; monitoring was performed during desensitization on days -14, -7, 0 and in a weekly basis until neutrophil engraftment. Desensitization strategies varied depending on center experience, immunofluorescence intensity, complement fixation and type of antibodies.

**Results:**

We identified a total of 20 haplo-HSCT in 19 patients performed with DSAs in 5 centers. 10 (53%) patients presented anti-HLA class I DSAs (6 of them with > 5000 mean fluorescence intensity (MFI)), 4 (21%) presented anti-HLA class II (1 with > 5000 MFI) and 5 (26%) presented both anti-HLA class I and II (5 with > 5000 MFI). 90% of patients received at least two treatments as desensitization strategy and all experienced a decrease of MFI after desensitization (mean reduction 74%). Only one patient who developed progressive increase of MFI after infusion developed GF. Desensitization treatments used included rituximab, immunoglobulins, therapeutic plasma exchange, incompatible platelets, buffy coat and immunosuppressors. Seventeen (90%) patients achieved neutrophil engraftment; one patient died before engraftment because of infection and one patient with class I DSAs developed primary GF despite an intensive desensitization. After a median follow-up of 10 months, OS and EFS were 60% and 58%, respectively, cumulative incidence of relapse was 5% and NRM was 32%.

**Conclusions:**

Despite the optimal strategy of DSAs desensitization remains unclear, the use of desensitization treatment guided by DSAs intensity kinetics constitute an effective approach with high rates of engraftment for patients with DSAs in need for an haplo-HSCT lacking an alternative suitable donor.

## Introduction

The expanding use of alternative donors, including unrelated donors, umbilical cord blood (UCB) and haploidentical transplant, has significantly increased the possibility of allogeneic hematopoietic stem cell transplantation (HSCT). In fact, the number of alternative donor transplants (especially haploidentical HSCT (haplo-HSCT)) increases every year (1). However, these modalities of HSCT have presented with new challenges in both donor selection and transplant strategy.

Donor-directed anti-human leukocyte antigen (HLA)- specific allo-antibodies (DSAs) are preformed IgG antibodies with specificity against HLA molecules not shared with the donor which are resistant to the standard conditioning regimen (2). Despite its significance mediating rejection was well-known in the setting of solid transplant, the first report in which the presence of complement-fixing anti-donor antibodies was associated with higher risk of graft failure (GF) was reported in 2002 in the setting of mismatched unrelated donor transplant (mMUD) (3). This finding was retrospectively confirmed in a larger cohort in mMUD transplants by the Atlanta group (2). In the subsequent years, the development of different strategies that made haplo-HSCT feasible, including the use of high-dose post-transplant cyclophosphamide pioneered by Luznik et al. (4, 5), led to an increased experience with patients with DSAs lacking alternative suitable donors. The first retrospective report from the MD Anderson group showed a high incidence of GF associated to the presence of DSAs in haplo-HSCT (6) and also the relationship between anti-DPB1 DSAs and GF in the setting of matched unrelated donor (MUD) transplant (7). Regarding UCB transplant, the relationship between DSAs and GF has been described in both single (8, 9) and double cord blood transplant (10). More recently, the Beijing group have reported the relationship of DSAs not only with GF but also with poor graft function.

Consequently, the screening for DSAs before MUD, mMUD, haplo and UCB transplant becomes mandatory (11). Among those, DSAs are more frequently reported in the haplo-HSCT setting, due to both higher grade of mismatch and the possibility of alloimmunization after pregnancies against offspring antigens in the case of female recipients, with a prevalence between 10 and 20% and that can reach more than 80% in female with multiple pregnancies (12).

Currently, the gold standard for the detection of DSAs is based in solid-phase immunoassays (SPI) using the Luminex^®^ platform (13). SPI are non-quantitative assays in which the patient serum is incubated with a solid-phase coupled to different exposed HLA-molecules; if there is presence of antibodies, immunofluorescence is detected. Despite this is a non-quantitative technique, this approach offers a result of immunofluorescence intensity (MFI) of the antibodies that has been related to their clinical significance (14). Moreover, the technique has also been modified to detect complement-fixing (C1q+ or C3d) antibodies that are specially clinically relevant, as reported to by Ciurea et al. (15)Thus, the increasing sensitivity of this technique has led to the concept of “virtual crossmatch” that have significantly decreased the use of conventional crossmatch tests (13).

As consequence, once DSAs are identified, an alternative donor against whom the patient has no DSAs should be looked for. However, due to the progressively smaller size of families and ethnical disparities that hinder the selection of a matched related or unrelated donor, alternative suitable donors are not always a possible solution, and hence, DSAs desensitization strategies become an option (16). During the last years, multimodal desensitization strategies have been reported by different groups (11, 12, 15–17), based on those developed for solid organ transplantation, including a combination of strategies that remove (therapeutic plasma exchange, TPE) or neutralize (incompatible platelet or buffy coat transfusion) preformed antibodies, reduce the production of antibodies (rituximab (RTX), bortezomib or immunosuppressive therapies), and inhibit the complement cascade (IV immunoglobulins, IVIG). The combination of these approaches can vary depending on the MFI intensity and the capacity of binding complement of the DSAs. The incidence of primary GF after desensitization has been reported in small case series and appear to be lower than 10% (18). However, there is a lack of studies comparing the different combination strategies and usually the procedure depends on center policies and accessibility as well as retrospective center-experience.

Despite several strategies have been reported, only one consensus guideline have been published by the EBMT in 2018, that includes several strategies (12). Thus, centers have used different approaches derived mainly from the cumulative experience with HSCT with haploidentical donors. The aim of this study was to report the experience of the Madrid Group of Hematopoietic Transplant (GMTH) with patients with DSAs undergoing haplo-HSCT.

## Patients and Methods

### Patients

Patients with DSAs transplanted with haploidentical donors in centers from the GMTH between January 2012 to June 2020 were included in the study. Disease and transplant characteristics were collected from the electronical registries of each hospital. The study was approved by the ethical committee of the Hospital General Universitario Gregorio Marañón, and all patients signed informed consent.

### HLA Typing and DSA Identification

According to the current recommendations, HLA typing was performed at high/intermediate resolution by DNA-based techniques (19). Haploidentical donors were all familiar donors sharing one single HLA haplotype. All donor and recipient HLA determinations were performed in two different samples. Patient sera was collected from clotted samples to perform Luminex ^®^ SPI IgG single antigen tests (Lifecodes, Immucor, USA), that cover HLA-A, B, C, DRB1, DRB3, DRB4, DRB5, DQA1, DQB1, DPA1 and DPB1 (13) antigens. In patients in whom antibodies were detected, complement-fixing antibodies (C3d) techniques to characterize the antibodies functionality were performed in the last period (2018-present). IgG MFI values between 1000 and 5000 were considered low, between 5000 and 10000 intermediate and >10000 high as considered by the EBMT consensus (12). C3d MFI were classified as negative or positive.

### DSA Monitoring

DSA screening was performed once the mismatched HSCT was indicated and repeated in a monthly basis in patients with identified DSAs. An additional sample was collected in all patients without DSAs within the month before starting conditioning regimen with independence of the previous antibody status, to discard a possible sensitization in patients with potential sensibilization events (including any transfusion). In patients with DSAs, monitoring was performed during desensitization on days -14, -7, 0 and in a weekly basis after the infusion of stem cells until myeloid engraftment was achieved.

### Desensitization Strategy

Strategies used varied according to center policies and experience, immunofluorescence intensity and type of antibodies (anti-HLA class I and/or anti-HLA class II). Treatments included RTX, IVIG, TPE, incompatible platelet transfusions, buffy coat transfusion and immunosuppressive agents (mofetil mycophenolate (MMF), tacrolimus and steroids).

### Pre and Post-Transplant Evaluation

Patients were stratified according to the disease risk index (DRI) (20). Pre-transplant comorbidities were recorded using the HCT–CI (21). Chimerism was determined by quantitative analysis of informative microsatellite DNA polymorphisms as previously described (22). Acute GvHD was scored according to the MAGIC criteria (23). Chronic GvHD was scored according to the NIH Consensus Development Project (24).

### Definitions

Myeloid engraftment was defined as an ANC of 0.5x10^9^/L or greater for 3 consecutive days. Platelet engraftment was defined as a platelet count of 20x10^9^/L or higher without transfusion support for 3 consecutive days. Patients who survived more than 30 days and failed to achieve myeloid engraftment were evaluated in an individual case-basis to discard a possible GF. Diagnosis of disease recurrence was based on clinical and pathological criteria.

### Statistical Analysis

Quantitative variables were expressed as median and either interquartile range (IQR) (25th and 75th percentiles) or range. Qualitative variables were expressed as frequency and percentage. Primary endpoints were rates of myeloid and platelet engraftment. Secondary endpoints included occurrence of aGVHD and cGVHD, relapse, NRM or death of any cause. Relapse, toxic death and second transplant due to GF were considered events. Estimates of event-free survival and overall survival were calculated using the Kaplan–Meier method. Cumulative incidence curves and competing risk regression were performed as alternatives to Cox regression for survival data in the presence of competing risks. In our case, competitor events for engraftment were toxic death, second transplant and any other occurrence that prevents the appearance of the event. For the cumulative incidence of myeloid, platelet engraftment and full donor chimerism, death and re-transplantation due to GF were considered competing events. NRM and relapse were considered competing events for each other, in addition to re-transplantation for both of them. Last update of the cohort was performed in August 2020. Except for the cumulative incidence, all calculations were made with SPSS (IBM, SPSS Statistics for Windows, Version 21.0. Armonk, NY, USA). Cumulative incidence was calculated with R Studio version 1.0.2. Due to the limited sample size, multivariate analysis to identify predictive factors for the outcomes were not performed.

## Results

### Patients’ and Transplant Characteristics

Between January 2012 and June 2020, 453 patients underwent an haplo-HSCT in the 5 centers in Madrid. Among those, a total of 19 patients with DSAs underwent transplant from a donor against whom DSAs were present, with a total of 20 HSCT performed between March 2016 and June 2020. The characteristics of the 19 patients and transplants are shown in **Table 1**. One patient underwent two haplo-HSCT procedures with DSAs. Eighteen patients (95%) were female, all of them with prior pregnancies (16% of them with 3 or more pregnancies). All patients had received multiple transfusions prior to transplant. 18 patients were diagnosed with hematological malignancies: 8 (42%) acute myeloid leukemia (AML), 3 (16%) high risk myelodysplastic syndrome (MDS), 3 (16%) acute lymphoid leukemia (ALL), 3 (16%) non-Hodgkin lymphoma (NHL) and 1 (5%) Hodgkin lymphoma. Of note, the remaining patient was diagnosed with severe aplastic anemia (AAS) refractory to both immunosuppressive therapy and thrombopoietin analog. Two patients (11%) had received a previous allogeneic HSCT without DSAs, one from a matched related donor (MRD) and the remaining from a different haploidentical donor; both relapsed after the first transplant and had an urgent indication of a second procedure. 18 patients (95%) lacked HLA identical related or unrelated donor, and haplo donors were selected according to levels of DSAs present in the recipient.

**Table 1:** Patients and transplant characteristics.

	Patients (n = 19)
Sex (female, %)	18 (95)
Age (median, IQR)	56 (49-61)
Diagnosis (n, %):	
• AML/MDS	11 (58)
• ALL	3 (16)
• Non-Hodgkin lymphoma	3 (16)
• Hodgkin lymphoma	1 (5)
• Severe aplastic anemia	1 (5)
Disease risk index	
• Intermediate	10 (53)
• High/Very High	8 (42)
• Not-applicable	1 (5)
• HCT-CI score (n, %)	
• 0-2	9 (47)
• ≥3	10 (53)
Sensitization events (n, %):	
• Polytransfusion only	1 (5)
• 1-2 pregnancies + polytransfusion	15 (77)
• 3-4 pregnancies + polytransfusion	3 (16)
Prior allo-HSCT (n, %)	2 (11)
Donor (n, %):	
• Sibling	5 (26)
• Children	13 (69)
• 2nd grade relative	1 (5)
Stem cell source peripheral blood (n, %)	19 (100)
Graft counts	
• CD34+ cells (x10^6^/kg) (median, range)	6.7 (4.9-15)
• TNC (x10^8^/kg) (median, range)	9.2 (4.5-13)
Conditioning regimen intensity (n, %)	
• Myeloablative	10 (53)
• Reduced intensity	9 (47)
CMV serostatus (n, %)	
• Donor and recipient positive	17 (90)
• Donor and recipient negative	1 (5)
• Donor negative, recipient positive	1 (5)
ABO incompatibility (n, %)	
• None	16 (85)
• Major	1 (5)
• Minor	2 (10)

We identified a total of 20 haplo-HSCT performed with DSAs in 19 patients. Data from the second haplo-HSCT of the patient with 2 procedures is not shown in this Table. IQR, interquartile range; AML, acute myeloid leukemia; MDS, myelodysplastic syndrome; ALL, acute lymphoid leukemia; TNC, total nucleated cell count.

All transplants were performed using peripheral blood as stem cell source. Median CD34+ cells in the graft were 6.7 x10^6^/kg (range 4.9-15). 10 patients (53%) received myeloablative conditioning: 8 received FluBux regimen (fludarabine (Flu) 40 mg/m^2^/day on days -6 to -3 plus 3 or 4 days of IV busulfan (Bux) 3.2 mg/kg/day on days -6 to -3), one received VP16 + total body irradiation (TBI), and the remaining was conditioned with TBF regimen (Flu, Bux, TT) with thiotepa (TT) 5 mg/kg/day (2 days). Nine patients (47%) received reduced intensity conditioning regimen with a modified Baltimore protocol consisting of Flu 30 mg/m2/day days -6 to -2, cyclophosphamide (Cy) 14.5mg/kg/day on days -6 and -5 and Bux 3.2 mg/kg/day either one or two days on days -3 and -2. In addition to a reduced intensity conditioning regimen, the patient diagnosed with aplastic anemia received thymoglobulin 2 mg/kg/day on days -6, -5 and -4.

All patients received GVHD prophylaxis with high dose post-transplant cyclophosphamide 50 mg/kg/day on days +3 and +4 together with mofetil mycophenolate 10 mg/kg/day and either tacrolimus (12 patients, 63%) or cyclosporine A (7 patients, 37%) since day +5.

### DSA Characteristics, Kinetics, and Management

Among HLA specificity and MFI, 10 patients (53%) presented only anti-HLA class I DSA (6 of them with MFI over 5000), 4 patients (21%) presented only anti-HLA class II DSAs (1 of them with MFI over 5000), and the remaining 5 (26%) presented DSAs against both anti-HLA class I and II antibodies (all of them over 5000 MFI) (**Table 2**). Complement fixation techniques were performed in 6 patients, 3 of them with both anti-HLA class I and II antibodies and MFI >5000, and 3 with only anti-HLA class I, of whom 1 had MFI <5000. All of them showed C3d fixation and were considered positive.

**Table 2:** Donor specific antibodies: characteristics, kinetics and management.

	Patients (n = 19)
Baseline DSA characteristics (n, %)	
• DSA anti-MHC class I only	10 (53)
• *Intensity > 5000 MFI*	*6*
• DSA anti-MHC class II only	4 (21)
• *Intensity > 5000 MFI*	*1*
• DSA anti-MHC class I and II	5 (26)
• *Intensity > 5000 MFI*	*5*
After desensitization strategy, prior to infusion	
• Median reduction of intensity (median %, range)*	100 (20-100)
• Patients with detectable DSA at infusion (n, %)	3 (16)
• *Intensity > 5000 MFI*	*2*
Complement fixation techniques available (n, %)	6 (31)
• Positive C3d fixation	*6*
Increase after infusion (n, %)	1 (5)
• Intensity >5000 MFI	*1*
Desensitization treatment used (n, %):	
• Rituximab	17 (90)
• IGIV	13 (68)
• MMF	12 (63)
• TPE	11 (58)
• Incompatible platelet transfusion	9 (47)
• Buffy coat	6 (31)
• Tacrolimus	5 (26)
• Steroids	1 (5)

We identified a total of 20 haplo-HSCT performed with DSAs in 19 patients. Data from the second haplo-HSCT of the patient with 2 procedures is not shown in this Table. DSA, donor-specific antibodies; MHC, major histocompatibility complex; MFI, immunofluorescence intensity; IGIV, intravenous immunoglobulin; MMF, mofetil mycophenolate; TPE, therapeutic plasma exchange. *mean 74%.

All patients with DSAs received desensitization therapy. Desensitization treatment used were weekly RTX 375 mg/m^2^ in 17 patients (90%) (median doses 3.5 doses, range 1-6, administered weekly starting from day -35), IVIG 0.4mg/kg/day in 13 (68%) (median 5 days, range 3-10; 10 patients received IVIG during conditioning regimen during days -6 to -2, and 3 patients started administration 2 to 4 weeks before conditioning regimen was initiated), MMF 5-10mg/kg/bid in 12 (63%) (median 21 days, range 14-28, starting 2 to 4 weeks before the infusion date and until day -2), TPE in 11 (58%) (median 3 sessions, range 1-10; 6 patients started 2 to 3 weeks before conditioning regimen was initiated, and 5 received TPE during conditioning chemotherapy), incompatible platelet transfusion only if class I DSAs were present on days -1 and/or 0 in 9 (47%, median 3 pools, range 1-5), buffy coat only if class II DSAs were present on day -1 in 6 (31%), tacrolimus 0.06mg/kg/day in 5 (26%) (3 patients starting 2 to 4 weeks before infusion, and 2 patients on days -6 to -2), and dexamethasone in 1 patient (5%). Patients with MFI >5000 (n=12) underwent a more intensive desensitization including RTX (all cases) plus an immunosuppressor (11 patients, 92%), IGIV (10 patients, 83%), platelet or buffy coat transfusion (10, 83%), and TPE (8, 67%). Among the 7 patients with <5000 MFI, regimens used were less intensive, 5 received a combination of RTX with either IVIG, buffy coat or immunosuppressor +/- TPE, one received only TPE, and the remaining received transfusion of incompatible platelets. Detailed description of desensitization therapies and outcomes are included in the Supplementary material (**Supplementary Table 1**).

All patients experienced a decrease of DSAs intensity after desensitization strategies, with a mean reduction of 74% (median 100%, ranging from 20 to 100% of reduction). Three patients (16%) had detectable DSAs at day 0 prior to infusion, 2 of them with MFI over 5000. Only one patient (5%) experienced a progressive increase of immunofluorescence after infusion since day 0 and developed primary GF requiring a second salvage HSCT.

### Engraftment and Chimerism

**Figure 1:**
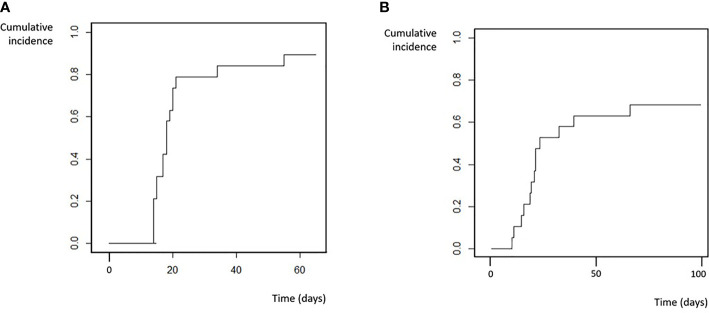
Engraftment. **(A)** Neutrophil. **(B)** Platelets.

Seventeen (90%) patients from the cohort achieved myeloid engraftment in a median of 18 days (range 14-55, IQR 15-21 days). One patient (5%) died at day 21 because of a severe infection without engraftment, and the remaining patient (5%) experienced primary GF and underwent a second transplant. Cumulative incidence of neutrophil recovery at day 28 was 79% and at day 60 was 89% (**Figure 1**). Fourteen (73%) of the patients achieved platelet engraftment in a median of 31 days (range 14-292, IQR 22-94). Three (16%) of the patients died because of toxic complications before platelet engraftment, and 2 (10%) patients had not achieved platelet engraftment at last follow-up (3 and 9 months post-HSCT). There were no secondary GF events.

Full-donor chimerism was achieved in 14 patients of 17 evaluable patients by day +30 (30-day cumulative incidence of 68%). Median time to full-donor chimerism achievement in peripheral blood was 25 days (range 14-50, IQR 18-33).

The patient who developed primary GF was a 63-year old female with 2 prior pregnancies, diagnosed with high risk MDS (excess blasts type 2 MDS, without myelofibrosis) without prior polytransfusion who received a myeloablative haplo-HSCT from her brother against whom she presented anti-class I DSAs against locus HLA-A*32 with 14000 MFI, and C3d fixation detected prior to desensitization therapy. Desensitization included weekly RTX (3 doses), MMF plus tacrolimus for 4 weeks, IVIG and TPE (5 days) and transfusion of incompatible platelets prior to stem cells infusion. Despite initial reduction of DSAs intensity of 82% at infusion, antibodies were present at 2500 MFI at stem cell infusion, and a progressive increase on MFI after day 0 over 5000 MFI at week 3 and over 10000 at last determination were detected. No evidence of myeloid engraftment was detected at day 30 and the patient showed autologous reconstitution in the chimerism study performed at day 30 (99% receptor). A boost of selected CD34+ cells from the same donor was infused on day +37 with no result, hence a second salvage HSCT from a different donor was required (2^nd^ grade relative). Anti-class II DSAs were also present against this donor (this procedure was the 20^th^ haplo-HSCT with DSAs of this cohort). Desensitization was performed again resulting in a decrease of MFI and achievement of engraftment and full donor chimerism from the 2^nd^ donor. However, the patient suffered an early death due to infection (septic shock) after 2^nd^ transplant.

### Survival, Relapse, and Non-Relapse Mortality

**Figure 2:**
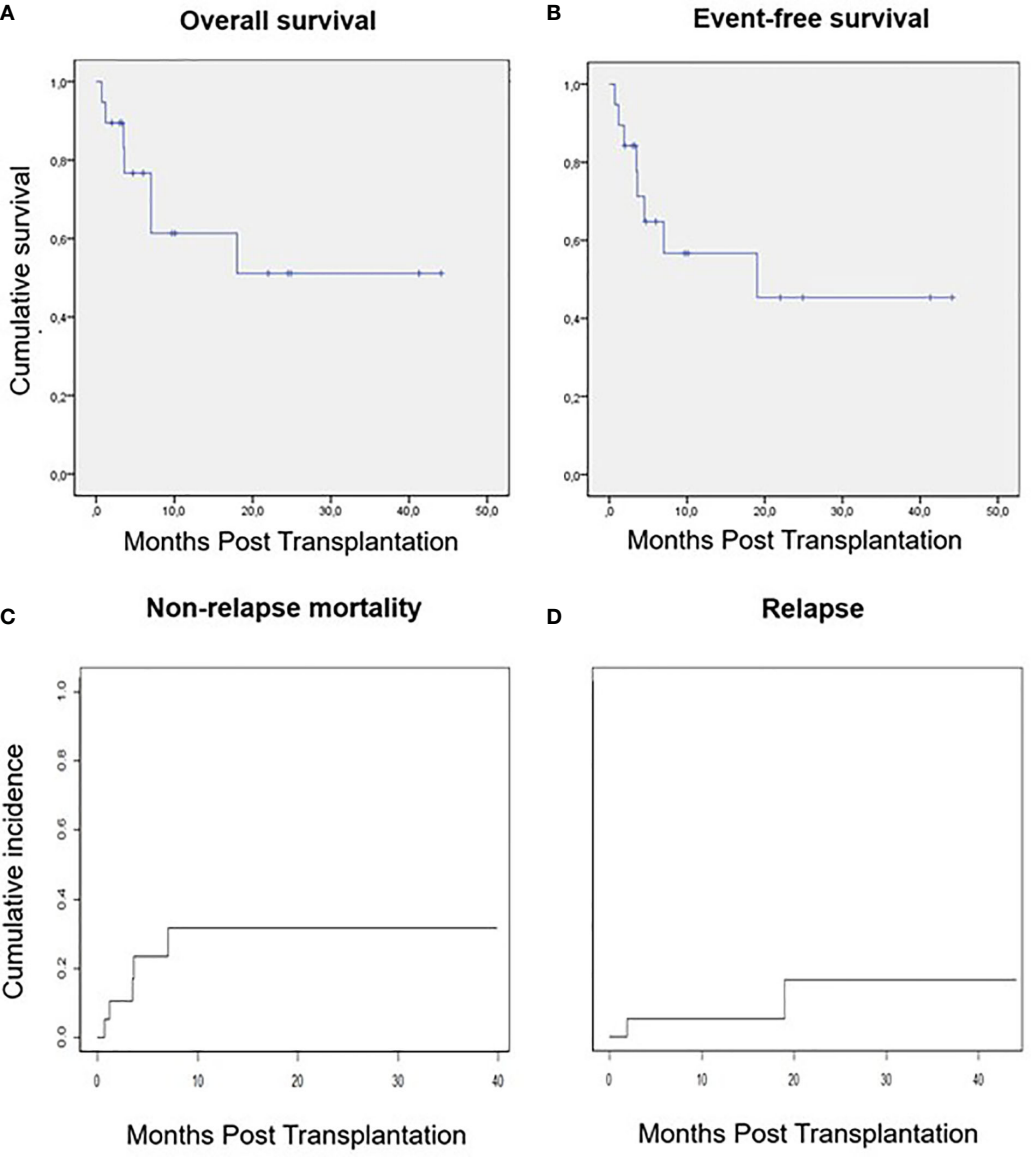
Survival, relapse and non-relapse mortality. **(A)** Overall survival. **(B)** Event-free survival. **(C)** Non-relapse mortality. **(D)** Relapse.

After a median follow-up of 10 months (range 3-44 months), the 10-month overall survival (OS) and event-free survival (EFS) were 60% (95% CI 57.5-62.5) and 58% (95% CI 55.5-60.5) respectively (**Figure 2**). Cumulative incidence of relapse (CIR) at 10 months was 5% and estimated CIR at 2-years was 16% (estimated at 2-years 16%). Cumulative incidence of non-relapse mortality (NRM) at 10 months was 32% (estimated at 2-years 32%).

A total of 7 (37%) patients died during the study period. One patient (5%) because of primary GF, three patients (16%) due to infection, 1 of them in the immediate post-transplant period at day 21 because of a gram-negative bloodstream infection (BSI) and 2 after day 100, one of them because of infection in the context of immunosuppressive therapy required for steroid-refractory grade IV acute graft-versus-host disease (aGVHD). Two patients (10%) died before day 100 because of endothelial complications, one with transplant-associated thrombotic microangiopathy (TA-TMA) and the remaining with sinusoidal obstruction syndrome (SOS). Only one patient died due to relapse of the underlying disease.

### Toxicity

Severe infections occurred before day 30 in 5 patients (26%) including 7 episodes of BSI in 4 patients, and one invasive fungal infection. After engraftment, 2 additional patients died due to infection as reported, one at month 7 because of septic shock secondary to *Pseudomonas aeruginosa* BSI, and the remaining also related to *P. aeruginosa* infection in the context of intensive immunosuppressive therapy for GVHD at month 3. 15 patients (79%) experienced at least one cytomegalovirus (CMV) reactivation in the first 6 months after HSCT.

Endothelial complications were diagnosed in 4 patients (21%). Two (10%) of them met criteria of TA-TMA, one diagnosed with AML and the other with AAS; both patients had received reduced intensity conditioning regimens. Both presented anti-HLA class I together with anti-class II DSAs and had received buffy coat and RTX as common treatments (one of them with a combination of IGIV, MMF, TPE and incompatible platelets). One patient received eculizumab with complete response of TA-TMA, and the remaining received eculizumab, defibrotide and TPE achieving PR but developed an abdominal origin septic shock and died. Two patients (10%) developed SOS, one case was mild and related to previous treatment with inotuzumab ozogamicin, with complete response of the process with supportive treatment; the other patient (diagnosed with AML) developed very severe SOS after a myeloablative HSCT requiring ICU admission, defibrotide and transjugular intrahepatic portosystemic shunt, and despite its initial partial response finally died due to intracranial hypertension.

Neurological complications were reported in 3 (16%) patients. Two patients (10%) developed posterior cord syndrome during CMV reactivations, without evidence of infection in the cerebrospinal fluid (CSF) and without specific findings in magnetic resonance imaging study. Both patients achieved near complete response after IVIG treatment, rehabilitation therapy and control of CMV viremia. One patient (5%) developed Guillain-Barré-like demyelinating polyneuropathy of unknown origin achieving complete response after IVIG treatment. All these 3 patients had received desensitization therapy with RTX, IVIG and MMF; 2 of them received incompatible platelets, 2 of them TPE and 1 of them buffy coat transfusion and tacrolimus.

### GVHD

The cumulative incidence of grade II-IV aGVHD at day 180 was 16% with one event of grade IV aGVHD accounting for a cumulative incidence of grade III-IV aGVHD of 6% at day 180. Cumulative incidence of chronic GVHD at 10-months was 16%, with 6% cumulative incidence of moderate-severe cGVHD. Of the 12 surviving patients at last follow-up, 2 had developed moderate cGVHD, one of them achieving partial response and the remaining complete response with steroid therapy.

## Discussion

The development and improvement of strategies that allowed the extended use of haploidentical HSCT has increased the pool of patients in need of an allogeneic HSCT that can benefit from this procedure (25, 26). However, the higher incidence of GF in haplo-HSCT compared to HLA-identical transplants due to the high degree of mismatch is still a challenge in some of these platforms (27). DSAs play a major role as a risk factor for GF in haplo-HSCT (6, 15). Consequently, screening for the detection of DSAs is mandatory in mismatched transplant recipients and an alternative donor against whom the patient has no sensitization should be searched, including other haploidentical related donors, UCB and/or 9/10 mMUD. However, those alternatives are not available in all transplant centers, and there are situations that do not admit the waiting time to identify an alternative donor, such as advanced leukemias or GF. In fact, it has been largely known that in these situations there is a negative impact on survival if the transplant is delayed (28). Thus, a strategy for monitoring and desensitization should be applied in those situations in which haplo-HSCT with DSAs is the best option. Desensitization strategies are mostly based in the accumulated experience in solid organ transplants, and the experience with them has mostly been provided by the groups with larger experience with haplo-HSCT. The EBMT has published guidelines reviewing the evidence for both the diagnosis and management in this specific situation (12). However, during the last years, centers had usually adopted strategies based in their own experience and the accessibility of treatments, designing an individualized approach for each patient.

Herein we describe the management and outcomes of patients undergoing an haplo-HSCT with DSAs over a 5-year period in the Madrid region. All centers performing haplo-HSCT in Madrid were asked about their experience and 20 transplant procedures with DSAs in 19 patients were identified. Of note, all HLA typing and DSAs study were centrally performed and the reports of DSAs kinetics and evolution during desensitization were received at the clinics within 48 hours in order to contribute to the clinical decision making. Similarly to other experiences, combinations of different treatments depending on DSAs class and intensity, and guided by the results of MFI reduction, were used. In spite of the lack of a consensus protocol, most of them included RTX, IVIG, an immunosuppressor, TPE and platelet or buffy coat transfusion, and recipients with less than 5000 MFI received a less intensive approach. This accomplished myeloid engraftment in all evaluable patients (90%) but one, accounting for a primary GF rate of 5%, which is comparable to that obtained in the whole cohort of haplo-HSCT (453 patients with a primary and secondary GF rates of 4% and 1%, respectively). It is noteworthy that this patient was the only one in the cohort that experienced a progressive increase of DSAs intensity after infusion, supporting the need of post-infusion monitoring and the predictive value of these monitoring for GF prediction, as previously reported (17), and the need of additional treatment in this setting.

The combination of desensitization strategies described in this study varied depending on the type of DSAs (anti-HLA class I vs. II) and their intensity, with a global consensus for a more aggressive approach in patients with MFI over 5000. Currently, there is no evidence favoring one approach over the other and the results in terms of engraftment obtained should be compared to those obtained by the largest series reported by the MD Anderson Cancer Center (MDACC) and the Baltimore group. As reported in the EBMT guidelines, the MDACC had analyzed the outcomes of 48 patients with DSAs of whom 26 were treated with TPE, rituximab, IVIG with or without buffy coat for patients with anti-class II DSAs. Patients with MFI under 5000 were not treated, and 2 out of 6 patients in this group tested for C1q were positive and failed to engraft. Among the treated patients, overall engraftment rate was 92% (12, 18). The Johns Hopkins group developed a protocol combining a combination of TPE, IVIG and immunosuppressive treatments (MMF/tacrolimus) starting 1 to 2 weeks before conditioning, and repeated TPE and IVIG if there was a peri-infusion rebound. With this approach, all 14 analyzed patients achieved engraftment by day +60. Of note, myeloid engraftment was delayed similarly to that reported in our study. Relapse rate in this study was high (50%) and has been postulated to be related the strong immunosuppression induced by the desensitization strategy (17).

**Figure 3:**
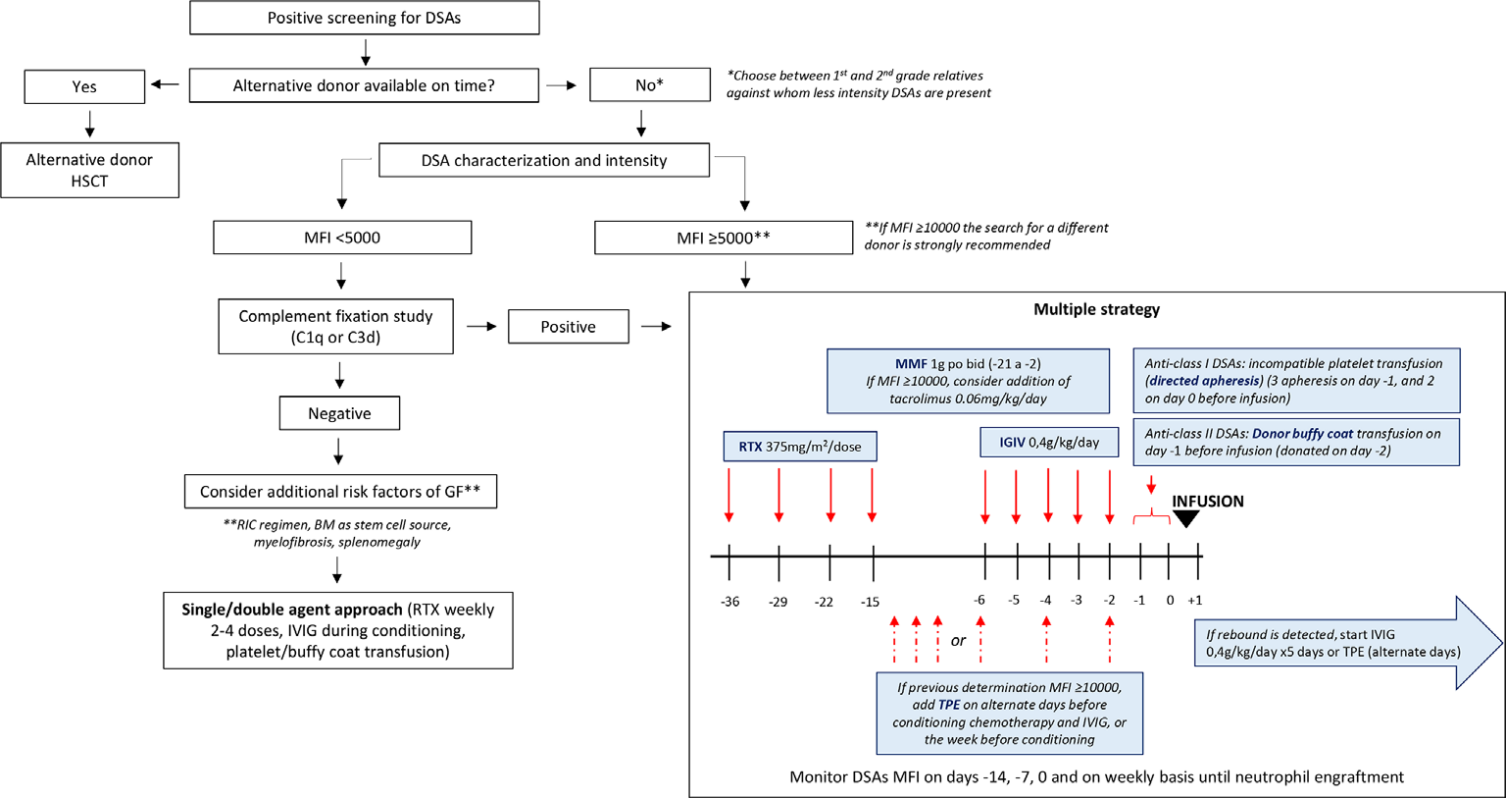
Proposed algorithm for the monitoring and management of DSAs in haplo-HSCT; MFI, mean fluorescence intensity; GF, graft failure; RIC, reduced intensity conditioning; BM, bone marrow; RTX, rituximab; IGIV, intravenous immunoglobulin; MMF, mofetil mycophenolate; TPE, therapeutic plasma exchange.

Our study includes a heterogeneous group of patients over a large period of years in which there were no consensus guidelines. Thus, its limitations include its retrospective nature, the limited number of patients, and the relative short follow-up. All patients received some desensitization therapy, even those who had <5000 MFI intensity. However, patients with MFI > 5000 and/or detection of C3b in those available underwent a more intensive desensitization approach including RTX and IVIG, an immunosuppressor and platelet or buffy coat transfusion in most cases and TPE in more than a half, in contrast with those with <5000 MFI, who received mostly only one or two treatments, including RTX in all but 2 cases. Thus, our approach in patients with low intensity DSAs contrasts with that proposed by the EBMT. There is a lack of evidence supporting one approach over the other. However, in these cases, complement fixation capacity of the DSAs together with additional classical risk factors of GF (use of bone marrow, reduced-intensity conditioning regimen, presence of myelofibrosis or splenomegaly) could guide the decision on whether desensitization could be needed. Based on our experience and the ones previously reported, we propose an algorithm of management of donor selection, DSA monitoring and desensitization in these patients (**Figure 3**), in which new strongly recommend the search for a different donor in patients with MFI ≥ 10000.

Despite engraftment rates (89% at day 60) were comparable to that reported by other groups, a delay of platelet engraftment and absence of platelet engraftment in 2 patients by day +100 was also observed. In those patients without platelet engraftment, antibodies anti-HPAa1 were discarded and platelet engraftment was achieved after day 100 using thrombopoietin analogues. The low incidence of relapse in our study (5% at month 10) could be explained by both the short follow-up and the relatively high NRM of 32% by month 10, with an overrepresentation of endothelial complications as compared to that reported in haplo-HSCT series (29, 30). However, with our limited sample size, conclusions on whether the endothelial damage is related to DSAs, their management or other not-related factors cannot be postulated. Causes of toxicity and NRM in these peculiar setting have not been extensively described in previous reports.

Donor selection in patients with DSAs is a challenging situation. The first option in these cases should be the search of a donor against whom DSAs are absent. However, due to the progressive smaller size of families, the probability of finding another suitable sibling is usually low. One possible alternative could be the search of haploidentical donors among second grade relatives (31); however, DSAs could also recognize HLA molecules present in these donors. The use of UCB should also be considered in these situations. However, DSAs can also recognize antigens present in cord blood units, and UCB transplant has peculiarities (including delayed engraftment) that could be limit its results in this peculiar setting. In fact, a recent randomized clinical trial has demonstrated the superiority of haplo-HSCT in terms of overall survival as compared with double cord transplant (32). In the recent years, the Baltimore group has pioneered an approach using HLA mismatched unrelated donor sharing ≥5/10 but <10/10 HLA alleles (HLA-A, -B, -Cw, -DRB1, -DQB1), using bone-marrow stem cells and a PTCy-based regimen with a calcineurin inhibitor and rapamycin (33). This approach, which has shown encouraging results in terms of engraftment, could also been used in patients presenting DSAs against familiar haplo donors who fail to decrease DSAs intensity after desensitization. Recently, a successful case of a patient who fail to DSAs desensitization and was transplanted with a PTCy based approach from a 5/10 mMUD against whom no DSAs were present has been reported (34). In these cases, the search of the unrelated donor becomes a challenging but encouraging approach.

In conclusion, despite the optimal strategy for DSAs sensitization remains unclear, the use of desensitization treatment guided by DSAs intensity kinetics constitute an effective approach for patients with DSAs against in need for an haplo-HSCT lacking another suitable donor, even in non-malignant disorders. This approach showed high rates of engraftment and comparable survival to that reported in the setting of haplo-HSCT, and most importantly, allowed to carry on with the procedure of allogeneic HSCT in a timely manner without delays. Further studies should address whether this approach has similar results for patients with DSAs to that obtained with unrelated, < 9/10 mismatched donor transplant, against whom no DSAs are present. Moreover, further studies should also address which desensitization approach is optimal depending on DSAs intensity, complement fixation and dynamics to prevent both overtreatment of patients and GF.

## Data Availability Statement

The raw data supporting the conclusions of this article will be made available by the authors, without undue reservation.

## Ethics Statement

The studies involving human participants were reviewed and approved by COMITÉ de ÉTICA DE LA INVESTIGACIÓN con MEDICAMENTOS del HOSPITAL GREGORIO MARAÑÓN. The patients/participants provided their written informed consent to participate in this study. Written informed consent was obtained from the individual(s) for the publication of any potentially identifiable images or data included in this article.

## Author Contributions

Conception and design: MK, JV, and RB. Provision of study materials or patients: All authors. Collection and assembly of data: RB, JAr, LS, IS-V, AC, and MC. Data analysis and interpretation: All authors. Manuscript writing: RB, JV, and MK. All authors contributed to the article and approved the submitted version.

## Conflict of Interest

The authors declare that the research was conducted in the absence of any commercial or financial relationships that could be construed as a potential conflict of interest.

## Supplementary Material

Supplementary Table 1 Management of DSA and results according to HLA class and intensity. DSA, donor specific antibodies; Pt, patient; AML, acute myeloid leukemia; ALL, acute lymphoid leukemia; NHL, non-Hodgkin lymphoma; HL, Hodgkin lymphoma; HR-MDS, high risk myelodysplastic syndrome; ATCL, adult T-cell leukemia/lymphoma (HTLV1); IS, immunosuppression; CR, complete response; PR, partial response; SD, stable disease; RIC, reduced intensity conditioning; MAC, myeloablative conditioning; RTX, rituximab; IGIV, intravenous immunoglobulins; TPE, therapeutic plasma-exchange; MMF, mofetil mycophenolate; FK, tacrolimus; SOS, sinusoidal obstruction syndrome; TA-TMA, transplant-associated thrombotic microangiopathy; aGVHD, acute graft-versus-host disease; cGVHD, chronic GVHD.
