# Supplementary material for: Management of Donor-Specific Antibodies in Haploidentical Transplant: Multicenter Experience From the Madrid Group of Hematopoietic Transplant
Source: Front Immunol. 2021 May 19;12:674658. doi: 10.3389/fimmu.2021.674658 (PMC8170127; doi:10.3389/fimmu.2021.674658)
Supplement: Supplementary Table 1 — Management of DSA and results according to HLA class and intensity. DSA, donor specific antibodies; Pt, patient; AML, acute myeloid leukemia; ALL, acute lymphoid leukemia; NHL, non-Hodgkin lymphoma; HL, Hodgkin lymphoma; HR-MDS, high risk myelodysplastic syndrome; ATCL, adult T-cell leukemia/lymphoma (HTLV1); IS, immunosuppression; CR, complete response; PR, partial response; SD, stable disease; RIC, reduced intensity conditioning; MAC, myeloablative conditioning; RTX, rituximab; IGIV, intravenous immunoglobulins; TPE, therapeutic plasma-exchange; MMF, mofetil mycophenolate; FK, tacrolimus; SOS, sinusoidal obstruction syndrome; TA-TMA, transplant-associated thrombotic microangiopathy; aGVHD, acute graft-versus-host disease; cGVHD, chronic GVHD. [file Table_1.pdf]

Supplementary Table 1. Management of DSA and results according to HLA class and intensity

			Patient and transplant characteristics				DSA						Outcomes, complications and follow-up						
			Patient	Indication	Conditioning	Donor	Highest initial MFI	C3d fixation available	Management	% Reduction (day 0)	MFI >5000 (day 0)	Increase after day 0	Engraftment	Endothelial complications	Infection	Neurological events	GVHD (II-IV / mod-severe)	Status	Follow up
DSA Anti-MHC I	MFI <5000	Pt 1	54-y, female	AML, CR1	MAC (FluBux4)	Male (son)	4500	No	RTX x4 doses, IVIG x5 days	100	No	No	Yes	No	Yes (severe pneumonia)	No	SR-aGVHD IV (day 40)	Dead (GVHD - infection)	3,6 months
		Pt 2	50-y, female	AML, CR1	MAC (TBF3)	Female (daughter)	2500	Yes (+)	TPE x1 session	100	No	No	Yes	No	No	No	No	Alive	41 months
		Pt 3	56-y, female	AML, CR1	MAC (FluBux4)	Male (son)	2700	No	Incompatible platelets	Unknown	Unknown	Unknown	Yes	No	No	No	No	Alive	10 months
		Pt 4	61-y, female	NHL, CR (previous auto-HSCT)	RIC (Bux2)	Female (daughter)	3400	No	RTX x1 dose, IVIG x3 days, TPE x5	100	No	No	Yes	No	Yes (bacteriemia)	No	No	Alive	10 months
	MFI ≥5000	Pt 5	49-y, female	AML, CR1	MAC (FluBux4)	Female (sister)	9000	No	RTX x2 doses, IVIG x5 days, MMF x2 weeks, incompatible platelets	20	Yes (7500 MFI)	No	Yes	No	No	Yes (posterior cord myelitis, CMV related?)	aGVHD II (CR) + mod cGVHD (CR)	Alive	44 months
		Pt 6	41-y, female	ALL, CR2 (previous allo-HSCT MSD)	RIC (Bux2)	Male (son)	14000	No	RTX x3 doses, IVIG x5 days, MMF x3 weeks, TPE x3 sessions, incompatible platelets	85	No	No	Yes	Mild SOS (previous inotuzumab)	Yes (bacteriemia)	Yes (demyelinating polyneuropathy)	No	Alive	25 months
		Pt 7	60-y, female	Sézary syndrome, SD	RIC (Bux2)	Male (son)	5000	No	RTX x4 doses, IVIG x 4 days, MMF x2 weeks, TPE x2 sessions, incompatible platelets	100	No	No	Yes	No	No	No	aGVHD II (CR)	Dead (relapse)	18 months
		Pt 8	60-y, female	HR-MDS, CR	RIC (Bux2)	Male (son)	5500	Yes (+)	RTX x4 doses, IVIG x 4 doses, Dexa 40 x4 days, MMF/FK x 2 weeks, TPE x2 sessions, incompatible platelets	100	No	No	No (early death)	No	Yes (bacteriemia)	No	No	Dead (infection)	0,7 month
		Pt 9	63-y, female	HR-MDS, CR	MAC (FluBux3)	Male (brother)	14000	Yes (+)	RTX x3 doses, IVIG x5 days, MMF/FK x4 weeks, TPE x5 sessions, incompatible platelets	82	No (2500 MFI)	Yes: increase up to >5000	No (2nd HSCT)	No	Yes (multiple bacteriemia)	No	No (censored)	Dead (after 2nd HSCT)	6 months
		Pt 10	41-y, female	ALL, CR1	MAC (FluBux4)	Male (brother)	11000	No	RTX x2 doses, IVIG x5 days, MMF/FK x4 weeks, TPE x5 sessions	100	No	No	Yes	No	No	No	No	Alive	2 months
DSA Anti-MHC II	MFI <5000	Pt 11	53-y, female	HL (previous allo-HSCT haplo)	RIC (Bux1)	Male (son)	3500	No	RTX x 3 doses, IVIG x5 days, MMF x2 weeks, FK x5 days	50	No	No	Yes	No	No	No	Moderate cGVHD (PR)	Alive (relapsed)	25 months
		Pt 12	56-y, female	AML, CR1	MAC (FluBux4)	Female (daughter)	3700	No	RTX x 1 dose, buffy coat	25	No	No	Yes	No	Yes (invasive fungal infection)	No	No	Alive	6 months
		Pt 13	66-y, male	HR-MDS, CR	RIC (Bux2)	Male (son)	4000	No	RTX x 1 dose, buffy coat	25	No	No	Yes	No	No	No	No	Alive	5 months
	MFI ≥5000	Pt 14	53-y, female	ALL, CR1	MAC (TBI-Cy)	Male (son)	13000	No	RTX x4 doses, TPE x2 sessions, MMF/FK x4 weeks	40	Yes (5500 MFI)	No	Yes	No	No	No	No	Alive	3 months
DSA Anti-MHC I + II	MFI ≥5000	Pt 15	57-y, female	ATCL (HTLV1), CR	RIC (Bux1)	Male (son)	11000 (HLA-I <5000)	Yes (+)	RTX x3 doses, IVIG x5 days, MMF x3 weeks, FK x5 days, TPE x4 sessions, buffy coat	100	No	No	Yes	No	No	Yes (posterior cord myelitis, CMV related?)	No	Alive	22 months
		Pt 16	42-y, female	IS-refractory Aplastic anemia	RIC (Bux2) + ATG	Male (brother)	11000	Yes (+)	RTX x4 doses, IVIG x5 days, MMF x3 weeks, TPE x3 sessions, incompatible platelets, buffy coat	80	No	No	Yes	Yes (TA-TMA)	Yes (bacteriemia)	No	No	Dead (TA-TMA)	3,5 months
		Pt 17	38-y, female	AML, CR1	MAC (FluBux4)	Female (cousin)	14000	Yes (+)	RTX x6 doses, IVIG x5 days, MMF x3 weeks, incompatible platelets, buffy coat	100	No	No	Yes	Yes (severe SOS)	No	Yes (non-filiated intracraneal HT)	No	Dead (SOS)	1 month
		Pt 18	69-y, female	AML, CR1	RIC (Bux2)	Male (son)	7000 (HLA-I <5000)	No	RTX x 1 dose, buffy coat	100	No	No	Yes	Yes (TA-TMA)	No	No	No	Aive	3 months
		Pt 19	63-y, female	AML, CR1	MAC (FluBux3)	Female (daughter)	25000	No	RTX x6 doses, IVIG x10 days, MMF x2 weeks, TPE x10 sessions, incompatible platelets	100	No	No	Yes	No	Yes (necrotizing fasciitis)	No	No	Alive	7 months
